# Amphiphilic Gold Nanoparticles: A Biomimetic Tool to Gain Mechanistic Insights into Peptide-Lipid Interactions

**DOI:** 10.3390/membranes12070673

**Published:** 2022-06-29

**Authors:** Ester Canepa, Annalisa Relini, Davide Bochicchio, Enrico Lavagna, Andrea Mescola

**Affiliations:** 1 Department of Physics, University of Genoa, Via Dodecaneso 33, 16146 Genoa, Italy; ester.canepa@fisica.unige.it (E.C.); relini@fisica.unige.it (A.R.); bochicchio@fisica.unige.it (D.B.); 2 CNR-Nanoscience Institute-S3, Via Campi 213/A, 41125 Modena, Italy

**Keywords:** thiol-protected gold nanoparticles, cell-penetrating peptides, antimicrobial peptides, cell membranes, lipid bilayers, non-specific interactions, spontaneous membrane translocation, molecular dynamics

## Abstract

Functional peptides are now widely used in a myriad of biomedical and clinical contexts, from cancer therapy and tumor targeting to the treatment of bacterial and viral infections. Underlying this diverse range of applications are the non-specific interactions that can occur between peptides and cell membranes, which, in many contexts, result in spontaneous internalization of the peptide within cells by avoiding energy-driven endocytosis. For this to occur, the amphipathicity and surface structural flexibility of the peptides play a crucial role and can be regulated by the presence of specific molecular residues that give rise to precise molecular events. Nevertheless, most of the mechanistic details regulating the encounter between peptides and the membranes of bacterial or animal cells are still poorly understood, thus greatly limiting the biomimetic potential of these therapeutic molecules. In this arena, finely engineered nanomaterials—such as small amphiphilic gold nanoparticles (AuNPs) protected by a mixed thiol monolayer—can provide a powerful tool for mimicking and investigating the physicochemical processes underlying peptide-lipid interactions. Within this perspective, we present here a critical review of membrane effects induced by both amphiphilic AuNPs and well-known amphiphilic peptide families, such as cell-penetrating peptides and antimicrobial peptides. Our discussion is focused particularly on the effects provoked on widely studied model cell membranes, such as supported lipid bilayers and lipid vesicles. Remarkable similarities in the peptide or nanoparticle membrane behavior are critically analyzed. Overall, our work provides an overview of the use of amphiphilic AuNPs as a highly promising tailor-made model to decipher the molecular events behind non-specific peptide-lipid interactions and highlights the main affinities observed both theoretically and experimentally. The knowledge resulting from this biomimetic approach could pave the way for the design of synthetic peptides with tailored functionalities for next-generation biomedical applications, such as highly efficient intracellular delivery systems.

## 1. Introduction

Peptide molecules have gained enormous potential in the field of nanomedicine due to their biochemical diversity, biocompatibility, biodegradability, low immunogenicity and varied biological activity *in vivo* [1]. Peptides of natural and synthetic origin have been used, *inter alia*, for cancer therapy, tumor targeting, as well as the treatment of bacterial infections and viral diseases [2,3,4,5,6]. They include bioactive peptides that are isolated from vegetables sources, plant food, marine species, venom components and other animal constituents [7,8]. More importantly, functional peptides possess innate physicochemical properties linked to intrinsic molecular and structural features that enhance their bioactivity and biomedical potential in a wide range of applications [6,9]. This is the case, for example, of therapeutic peptides that are able to passively enter cells by avoiding energy-driven endocytosis. Within endocytic vesicles, extracellular biomolecules are often degraded into their building blocks by hydrolytic enzymes—a fate that is extremely detrimental to their therapeutic value and on-target specificity [10,11].

Endocytosis-independent cell-penetrating activity relies on rapid and direct translocation across the cell membrane and is tightly regulated by non-specific hydrophobic and electrostatic interactions occurring between the major components of membrane bilayer and peptide structure. Amphipathicity and structural flexibility are two key factors in giving peptide molecules the propensity to enter cells spontaneously using this internalization pathway. Specifically, the presence of long and flexible amphiphilic side chains, such as lysine (Lys) or arginine (Arg), allows functional peptides to stably interact with the lipid bilayer by a mechanism known as ’snorkeling’ [12,13,14,15,16,17]. In this process, the amphiphilic side residues of the peptide progressively enter biological membranes, burying the hydrocarbon part of their structure in the apolar core of the lipid bilayer, while their charged amino groups snorkel towards the polar membrane-water interface. In addition to balanced hydrophobic and electrostatic effects at the bilayer interface [18,19,20,21,22], this mechanism is promoted by significant local membrane deformation [23,24] and conformational flexibility of amphiphilic peptide residues [25].

Despite the great biological potential of peptide-lipid interactions, the precise molecular events that mediate and regulate the encounter between membrane-interacting peptides and prokaryotic or mammalian cells are still poorly understood [26,27]. Most importantly, understanding the link between peptide structure (e.g., flexibility) and amphipathic properties at molecular resolution and the exact mechanism of interaction with cell membranes is a research priority in the arena. Still, this task is tremendously hampered by the heterogeneous chemical structure that characterizes both functional peptides and the cell membrane and further work needs to be performed. Synthetic nanomaterials with controlled and tailored flexibility and amphipaticity mimicking those of peptide molecules can be an extremely powerful resource to overcome this limitation. The interfacing of finely tuned nanomaterials with cell membranes can in fact provide an incredibly useful and reliable system for tackling the study of the physicochemical processes underlying peptide-lipid interactions.

Small (sub-10 nm) gold nanoparticles (AuNPs) protected by a mixed self-assembled monolayer of hydrophobic and hydrophilic thiols immediately stand out among synthetic nanomaterials for their ability to passively enter cells *in vivo* [28] and *in vitro* [29,30,31,32,33,34,35]. Gold is widely used as a scaffold to create ligand-protected NPs for biological applications, due to its remarkable stability, biocompatibility and electrical properties [36]. The gold core also enables finely controlled amphiphilic surface properties to be achieved by grafting a combination of apolar alkylthiols with a variable number of carbon atoms (usually greater than 8) and polar alkylthiols with a similar chain length and negatively or positively charged terminal groups (e.g., sulphonate or trimethylammonium ions) [37]. This mixture of amphiphilic ligands exhibits a peculiar snorkeling behavior very similar to that previously described for amphiphilic peptide side chains [38,39,40]. In addition to amphipathicity, another key feature of these ultrasmall AuNPs is the large portion of free volume available for each thiol ligand, which provides considerable surface conformational flexibility [40,41]. This allows the ligands to deform when entering the bilayer, favorably shielding their apolar alkyl chains within the hydrocarbon lipid tail region, while positioning the charged terminal moieties at the aqueous interface. As with amphiphilic peptides, non-specific electrostatic and hydrophobic effects are the main factors responsible for the stable binding of these NPs to membrane surfaces [38,39,40].

Inspired by this remarkable analogy, in this review we introduce a critical comparison of the membrane interaction effects induced by amphiphilic peptides and amphiphilic thiol-protected AuNPs. To analyze the topic from a molecular perspective, we will look separately at the impact of these interactions on key features of the cell membrane, such as the lateral phase separation and cholesterol-mediated fluidity. The membrane effects on the particles’ or peptides’ behavior, in particular the tendency to aggregate, will be also addressed. On the peptide side, we will discuss concrete examples of small molecules that share with amphiphilic thiol-protected AuNPs a high affinity for cell membranes, such as the well-known antimicrobial peptides (AMPs) and cell-penetrating peptides (CPPs) [26,42]. Both families possess an amphipathic structure comprising short-chain amino acid residues and a net charge due to a large amount of basic moieties such as Lys and Arg [26]. 

In order to focus on non-specific interactions mediated by electrostatic and hydrophobic contributions, our discussion will consider membrane environments devoid of energy-driven cellular processes. These membrane systems include biomimetic lipid membranes—such as lipid vesicles and supported lipid bilayers—which are widely exploited in parallel with *in vitro* cell research to gain mechanistic insights into peptide interactions at the cell interface [26]. Interestingly, the passive permeation of amphiphilic thiol-protected AuNPs has also been systematically observed in biomimetic lipid bilayers—both planar and curved—that exhibit fluid properties [40,43,44,45,46,47,48]. 

Overall, this review will present a thoughtful and novel perspective on the use of amphiphilic thiol-protected AuNPs as a suitable model to unravel and elucidate the molecular details of non-specific peptide-lipid interactions. In addition to understanding a wide range of peptide-mediated cellular processes, this advance in knowledge could be instrumental in designing peptides with tailored functionality for specific applications, e.g., for highly efficient intracellular delivery systems.

## 2. Amphiphilic Gold Nanoparticles and Amphiphilic Peptides: A Direct Route of Penetration into Lipid Membranes

**Figure 1:**
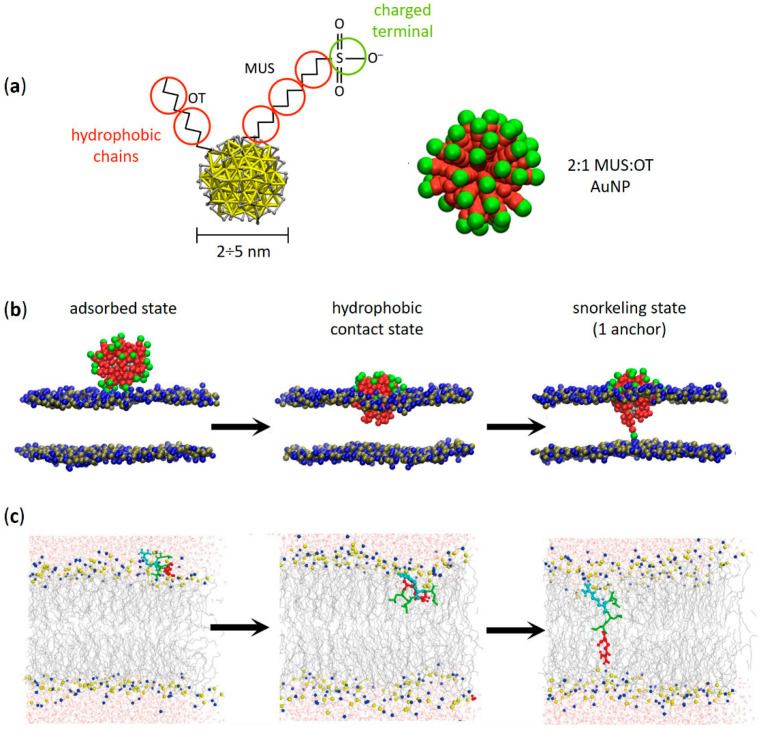
Amphiphilic AuNPs and amphiphilic peptides: similarities between their spontaneous penetration mechanism into lipid membranes. (**a**) Structure of an amphiphilic MUS:OT AuNP with its coarse-grained (CG) representation (2:1 MUS:OT ligand ratio). Red beads represent hydrophobic carbon groups, while green beads represent the charged MUS terminals. (**b**) Different stages of penetration of a MUS:OT AuNP into a 1-palmitoyl-2-oleoyl-glycero-3-phosphocholine (POPC) lipid bilayer obtained from CG molecular dynamics simulations. From left to right: adsorbed state, hydrophobic contact state, and snorkeling of the first MUS ligand which binds to the opposite leaflet. Eventually—through a sequential anchoring process—more and more MUS ligands are dropped leading to the fully snorkeled configuration of the NP-membrane complex. Lipid heads are blue (choline) and tan (phosphate), lipid tails and water are not shown. (**c**) Translocation process of an amphiphilic ‘Spontaneous Membrane-Translocating Peptide’ (SMTP) into a POPC lipid bilayer obtained from united atom bias-exchange metadynamics simulations. Specifically, the SMTP contains a LRLLR sequence composed of two Arg (R) and three leucines (L) residues. From left to right: SMTP located in the lipid head region, SMTP on its way towards the opposite leaflet, and final snorkeled configuration. The first Arg is shown in cyan, the second Arg is shown in red, and leucine hydrophobic residues are shown in green. Nitrogen and phosphorus atoms in the lipid head region are shown in blue and yellow, respectively. The lipid tails are shown as thin gray lines, while water is shown as red (oxygen) and gray (hydrogen) cylinders. (**a**,**b**) adapted with permission from Simonelli et al. [38]—Copyright © 2015 American Chemical Society. (**c**) reprinted with permission from Cao et al. [51]—Copyright © 2020 Elsevier B.V. All rights reserved.

Amphiphilic, thiol-protected, gold nanoparticles are known to be able to passively permeate the lipid bilayer of cellular or biomimetic membranes. Since their core is usually in the 2–5 nanometers range, it is extremely challenging to analyze their membrane penetration process by experimental means. In the last decade, several computational groups dedicated a massive effort toward elucidating the precise molecular mechanisms of the penetration into lipid membranes of AuNPs functionalized with 11-mercapto-1-undecanesulfonate (MUS) and 1-octanethiol (OT) (hereafter called MUS:OT AuNPs, Figure 1a). MUS:OT functionalization is one of the most popular and promising for the biomedical prospects of amphiphilic thiol-protected AuNPs, as it allows for spontaneous NP penetration into cells in a non-destructive way and the possibility of conjugating drug cargos for intracellular delivery [31,37,49]. MUS ligands terminate with a negatively charged group, which confers long-term colloidal stability to NPs in water and saline buffered solutions at pH 7.4 [50]. On the other hand, the hydrophobic OT ligands enhance the interaction between NPs and the membrane bilayer. Indeed, the combination of MUS and OT ligands allows for a passive penetration of the lipid bilayer while retaining a high solubility degree [50].

The picture of the penetration mechanism of amphiphilic MUS:OT AuNPs is now precise thanks to molecular dynamics (MD) studies employing all-atom and coarse-grained force fields [30,39,52,53], which revealed a multi-step process going through at least three different metastable stages (Figure 1b) [38]. In the first stage, the so-called “adsorbed state”, the NP adheres to the external surface of the bilayer. In this configuration, the NP charged terminal ligands interact with the zwitterionic headgroups of the membrane [38,54]. Coarse-grained simulations predict a reversible adsorption state, where the NP can detach and reattach later to the membrane [38]. At some point, the NP reaches the second stage of the penetration process, called the “hydrophobic contact state”, in which the hydrophobic moieties of the NP manage to contact the hydrophobic core of the membrane. The protrusion of one of the lipid tails to the polar head region of the bilayer is the molecular trigger of this configurational change [38,43,55]. As soon as the NP establishes the first hydrophobic contact, the NP ligands rearrange to maximize the interaction between the hydrophobic moieties of the ligands and the membrane core. The NP center of mass shifts toward the center of the bilayer, and the MUS charged terminals spread to contact the choline group of the membrane lipids [38]. From the hydrophobic contact state, one of the MUS ligands may cross the membrane core and attach to the lipid headgroups of the opposite leaflet: this is the “snorkeling state”, the third stage of the process [38,55]. During the translocation of a MUS ligand, simulations show a water channel formation [39] that is transient enough not to disrupt the membrane. In the snorkeled configuration, the NP center of mass further shifts towards the bilayer center; one after the other, the other MUS ligands eventually follow the first until the NP reaches a symmetric transmembrane configuration. As predicted by thermodynamic models, the latter is the most energetically favorable state of the NP-membrane complex [56,57,58,59].

It is worth noting that this picture of the penetration process, which depends on the presence of both hydrophobic and charged alkylthiol ligands in the NP functionalizing shell, can be considered valid also for non-MUS:OT AuNPs [37]. Indeed, amphiphilic thiol-protected AuNPs with different alkylthiol chain lengths, different surface charge sign and density, as well as different charged terminal groups, can spontaneously penetrate the lipid bilayer via ligand snorkeling [58,59,60,61,62]. For instance, deprotonated 11-mercaptoundecanoic acid (MUA) ligands can play the role of MUS ligands, replacing the SO_3_^–^ group with COO^–^ ions without substantial changes in the penetration mechanism described in Figure 1b [63]. The penetration mechanism remains the same even if the terminal group of the hydrophilic ligands is positively charged, like in the case of AuNPs protected by a mixture of OT and the cationic (11-mercaptoundecyl)-N,N,N-trimethylammonium (TMA) alkylthiol [46]. However, the free energy barrier relative to the snorkeling transition shown in Figure 1b can be reduced if the model accounts for partial protonation of negatively charged ligands at the membrane surface [63]. Indeed, the kinetics and thermodynamics of the process can vary significantly depending on the surface functionalization details, such as the ligand protonation state and ligand ratio [40,53,54]. The stability of the adsorbed state of MUS:OT AuNPs, for instance, depends on the NP surface charge density: the more MUS ligands, the longer the time of adhesion [38]. Interestingly, NPs with the same ligand shell composition but different patching patterns can have different penetration kinetics [30,38,40,53].

The ability of charged alkylthiol ligands to allow amphiphilic thiol-protected AuNPs to spontaneously penetrate the lipid bilayer recalls the same property of different types of membrane-interacting peptides, such as CPPs [64,65] and AMPs [29,66,67]. In numerous contexts, nature has designed peptides of these classes to penetrate the membrane effectively without the need for energy-activated endocytic processes. Usually, their transmembrane moieties are amphiphilic (i.e., Lys or Arg) and are effective when arranged in an α-helical structure [65,66]. In general, the reported membrane penetration process is quite similar to that depicted for MUS:OT AuNPs: an initial adhesion state, favored by the non-zero surface charge of the peptide, followed by a translocation, which requires local perturbation of the lipid bilayer (Figure 1c) [67]. Snorkeling effects similar to those observed for amphiphilic thiol-protected AuNPs have been reported for HIV-1 ‘Trans-acting Activator of Transcription’ (TAT) peptides, an Arg-rich CPP family that plays a direct role in the HIV disease process [68]; however, the translocation mechanism for TAT peptides involves transient pore formation [69]. The translocation mechanism for CPPs of the TP2 class has been studied in recent years both by experimental [70,71] and computational means [51,72] (Figure 1c), revealing that specific alternations of charged and hydrophobic amino acids may reduce the penetration barriers while causing hydrophilic pores or membrane deformations [72]. In other cases, the translocation mechanism does not require strong bilayer perturbation [70,73]. 

## 3. Similarities in the Lipid Membrane Interactions Effects of Amphiphilic Gold Nanoparticles and Amphiphilic Peptides 

### 3.1. Effects on Phase Separation

The complexity of the cell membrane—in terms of dynamic processes which locally modify the bilayer properties—is mainly related to the heterogeneity of its constituents (mostly lipids and proteins) and their lateral organization. In particular, the membrane lateral phase separation represents one of the main aspects of membrane complexity as it is involved in several relevant biological events, including trafficking, signal transduction and entry pathways of extracellular components. When evaluating membrane interactions with exogenous entities, be they nanoparticles, small molecules or drugs, lateral phase separation plays a central role that is mainly addressed through the use of different model lipid systems.

**Figure 2:**
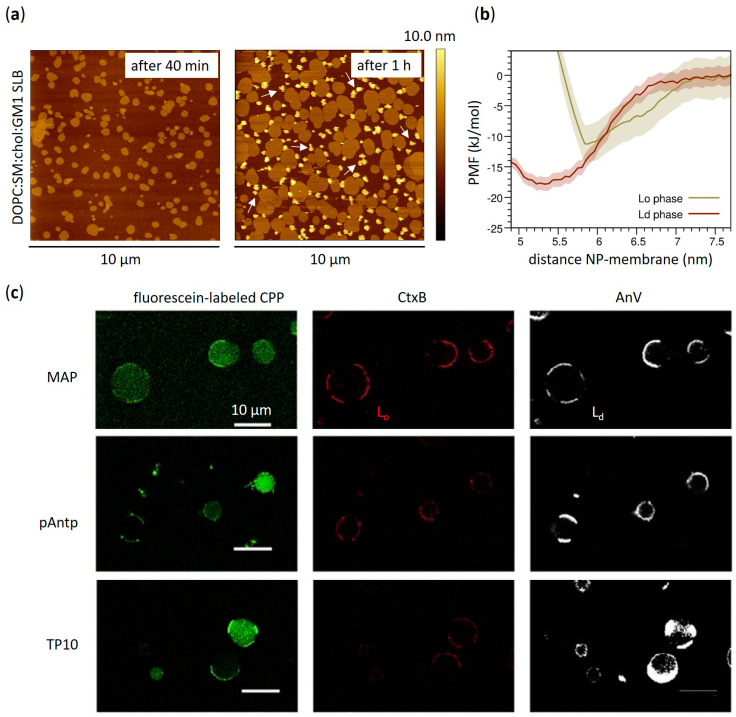
Amphiphilic AuNPs and amphiphilic peptides: a common affinity for the disordered domains of phase−separated lipid membranes. (**a**) Phase−separated SLBs containing 1,2-dioleoyl-sn-glycero-3-phosphocholine (DOPC), sphingomyelin (SM), cholesterol and ganglioside GM1 (63:31:1:5 molar ratio) imaged in liquid by atomic force microscopy (AFM) after addition of ~3 nm MUS:OT AuNPs (40 min and 15 h). After hours, large clusters of amphiphilic AuNPs (white arrows) slowly formed on the darker disordered phase and at the edges of the lighter (i.e., higher) ordered lipid domains. (**b**) Potential of mean force (PMF) profiles calculated for the adsorption of a single MUS:OT AuNPs on the surface of the L_d_ and L_o_ phase. The L_d_ phase, with a binding free energy of ~18 kJ/mol (~9 kBT), is favoured over the L_o_ phase (~11 kJ/mol, ~5 kBT). (**c**) Giant plasma membrane vesicles (GPMVs) derived from rat basophilic leukemia cells incubated at low temperature with three examples of fluorescein-labeled CPPs—i.e., MAP (model amphipathic peptide), penetratin (pAntp) and transportan 10 (TP10) (green). All these CPPs are amphipatic and contain Lys or Arg residues. L_o_ and L_d_ phases are labeled with AF594-labeled cholera toxin B subunit (CtxB, red) and AF647-labeled annexin V (AnV, pseudocolored as white), respectively. (**a**,**b**) contain images by Canepa et al. [44] reprinted with minor modifications under a CC BY-NC 3.0 license with permission from the Royal Society of Chemistry. (**c**) is reproduced and adapted with permission from Säälik et al. [81]—Copyright © 2011 Elsevier B.V. All rights reserved.

**Figure 3:**
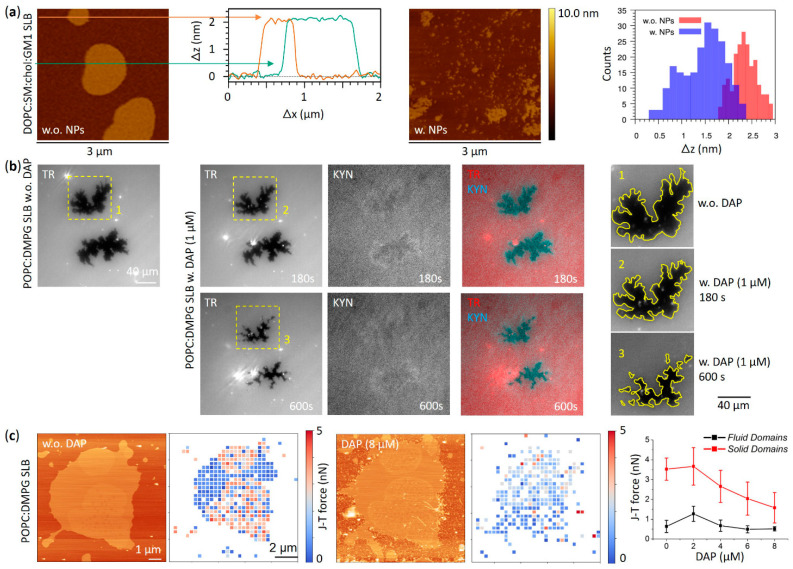
Perturbation of membrane ordered–disordered phase separation upon interaction with amphiphilic AuNPs and amphiphilic peptides. (**a**) Topographic AFM images showing fragmentation of ordered domains induced by ~3 nm MUS:OT AuNPs on phase-separated lipid bilayers containing DOPC:SM:chol:GM1 (63:31:1:5 molar ratio). Two height profiles of the phase-separated membrane without NPs are also reported. On the right: comparison of height difference distributions (Δz) between ordered and disordered domains before and after NP/membrane interaction. (**b**) Fluorescence images of the same region of a POPC:1,2-dimyristoyl-sn-glycero-3-PG (DMPG) 1:1 phase-separated SLB with PG-enriched ordered domains recorded before and after exposure to the lipopetide DAP (1 μM). Two solid ordered domains (dark regions) in a liquid disordered background (bright region) containing the fluorescence lipid probe DHPE-Texas Red (TR, 1%) are shown. The channel of kynurenine (KYN)—an intrinsically fluorescent DAP residue—is used to visualize the morphology of ordered domains since DAP strongly interacts with PG lipids. Overall, the SLB/DAP interaction induces an extensive size reduction of the solid domains equal to 59%. (**c**) Force spectroscopy analysis and topographic AFM images of the same POPC:DMPG 1:1 SLB before and after exposure to increasing DAP concentrations (0–8 μM). Jump-through (J–T) force maps (resolution 32 × 32 pixels) are reported next to each AFM image, together with the comparison of solid and fluid domains jump-through force upon increasing concentrations of DAP. (**a**) contains images by Canepa et al. [44] reprinted with minor modifications under a CC BY-NC 3.0 license with permission from the Royal Society of Chemistry. (**b**,**c**) are reproduced and adapted with permission from Mescola et al. [99]—Copyright © 2020 American Chemical Society. All rights reserved.

The coexistence of liquid disordered (L_d_) and liquid ordered (L_o_) domains at the nanoscale—these last with their densely packed chains similar to gel phase but endowed with much higher lateral mobility—has been demonstrated to strongly influence the interaction between amphiphilic AuNPs and lipid membranes. For example, Melby et al. have reported a preferential attachment of positively charged amphiphilic thiol-protected AuNPs to supported lipid bilayers (SLBs) containing L_o_ domains with respect to those counting single homogeneous phase [74]. Different scenarios are proposed to justify this behavior ranging from the preferential NP interaction with a specific phase, to the different mechanical properties of the bilayer responsible for guiding the NP partitioning, up to the role of the boundary between the two phases. Indeed, the phase boundary is commonly associated with factors that potentially drive the NP adsorption, such as the occurrence of unusual water structure or the enhanced permeability and the bilayer thickness variations [75,76,77,78]. The impact of lipid phase behavior on the adsorption of positively charged amphiphilic thiol-protected AuNPs has also been recently assessed by computational studies [79]. Interestingly, MD simulations by Sheavly et al. unraveled the physicochemical driving forces involved in the adsorption and partitioning of these AuNPs (4 nm core size) into phase-separated membranes, pointing to NP adsorption as a competitive mechanism between favorable NP-lipid interactions and the unfavorable curvature deformation of the bilayer [79]. Although theoretical approaches suggest a stronger interaction with lipid bilayers containing L_d_ domains due to their lower bending modulus, the measured free energy changes associated with the transport of a single amphiphilic AuNP through a phase-separated lipid bilayer revealed a minimum energy in correspondence of the phase boundary [79]. Such free energy minimum is reasonably attributed to the thickness disparity between L_o_ and L_d_ domains that enables favorable NP-lipid interactions without needing large curvature deformations. The effect of membrane phase separation on the absorption of negatively charged MUS:OT AuNPs was also assessed by Atukorale et al. comparing giant multilamellar vesicles (GMVs) composed of lipids having different melting temperatures (T_M_). In the case of high-melting lipids, no NP-membrane interaction was reported below T_M_, sign that amphiphilic thiol-protected AuNPs are excluded from the gel phase of GMVs [47]. Recently, the interplay between amphiphilic MUS:OT AuNPs and phase-separated membranes was further investigated by studying the interaction with multicomponent lipid bilayers mimicking the composition of the neural plasma membrane [44]. The latter naturally forms phase-separated domains due to the abundance of sphingolipids and in particular sphingomyelins, the variety of which can give rise to extremely complex phase behaviors [80]. Experimental evidence by atomic force microscopy (AFM) not only showed a preferential interaction between amphiphilic MUS:OT AuNPs and the disordered phase of model neuronal membranes (Figure 2a,b) but also revealed the suppression of lipid phase separation induced by AuNPs in a concentration-dependent manner (Figure 3a) [44]. Such effect has been interpreted using MD simulations, which successfully predicted the NP-induced phase separation destabilization; in addition to this in silico investigation, a simple thermodynamic model based on simulations allowed to identify the dominant driving force of the process under investigation. Specifically, the main NP effect is to reduce the lipid-lipid enthalpy gain due to phase separation by perturbing lipid-lipid interactions in the liquid disordered domains [44].

Analogous impacts on phase separation were also noticed as a consequence of the interaction between lipid membranes and the previously mentioned CPPs or AMPs. Generally, these kinds of peptide structures constitute a large family of both endogenous and synthetic molecules mainly composed by short-chain amino acid residues with different conformational arrangements [82,83]. Even if they typically follow disparate molecular pathways, they all share the spontaneous tendency to directly interact with cell plasma membrane as a first activation step [84,85,86,87,88,89,90]. With particular reference to phase separation, the preferential interaction of different types of CPPs with membrane domains of high fluidity has been reported (Figure 2c) [81]. This is, for instance, the case of penetratin, a positively charged Arg-based CPP able to bind negatively charged membrane components such as glycosaminoglycans and anionic lipids, and to effectively transport active molecules into the cell [91,92]. Similarly to amphiphilic MUS:OT AuNPs [44], it has been proved that L_d_ phase promotes the peptide-membrane interaction with respect to L_o_ phases [93,94], demonstrating the high relevance of membrane fluidity in regulating the interaction between exogenous molecules and lipid membranes. Further experimental evidence performed on both uni- and multi-lamellar vesicles with several experimental techniques showed also the penetratin tendency to induce lamellar phase separation and lipid rearrangements favouring the transitions to gel rippled phase along with de-packing of membrane polar head-groups [95]. Quite recently, it has been further established that Arg-rich CPPs can also directly enter the cells by inducing phenomena such as membrane multilamellarity and subsequent formation of a fusion pore [96]. There are also cases of CPPs that induce a higher lipid order, such as the specific case of the S4(13)-PV peptide: Cardoso et al. [97] focused on phase separation effects, revealing that destabilization of the membrane structure –without compromising membrane integrity—is at the basis of the lipid-driven and receptor-independent mechanism of cell entry of this peptide. In particular, in addition to the strong influence on thermodynamics revealed by the shifting of phase transition to higher temperature and the substantial structural changes which result in an enhanced lipid order, the promotion of lipid domains segregation has been as well detected as a consequence of the S4(13)-PV peptide-membrane interaction. Similar effects have been observed also on live membranes as confirmed by the formation and the stabilization of quasi-hexagonal domains in HeLa cell membranes [97]. The opposing tendencies of functional peptides to stabilize and de-stabilize lipid membrane phases have been also reported for some AMPs and, in some notable cases, even for the same peptide. This is the case of Gramicidin A, which has been found to selectively interact with disordered domains and to induce or prevent lateral phase separation depending on the membrane lipid composition [98]. However, these discrepancies in behavior must be sought in the high heterogeneity of the membrane compositions and experimental conditions examined, which give rise to multiple complex microenvironments, each with their own peculiar chemical and physical properties.

Similarities in the interaction effects on phase separation of amphiphilic AuNPs and amphiphilic AMPs have been reported as well. For instance, recent theoretical studies by Su et al. [100] revealed the tendency of four different AMPs—i.e., Magainin-2, BP100, MSI-103, and MSI-78—to selectively partition the L_d_ phase domains with respect to the L_o_ ones in a lipid system composed of both saturated and polyunsaturated phosphocholines and cholesterol. This is in strong analogy with what was observed in the interaction between amphiphilic MUS:OT AuNPs and the model neuronal plasma membrane exhibiting phase separation [44]. Furthermore, a preferential interaction with the domain interface was found for BP100, MSI-103, and MSI-78 AMPs, a feature further reminiscent of the behavior of amphiphilic thiol-protected AuNPs reported by Sheavly et al. [79]. Curiously, it has been demonstrated that all these AMPs induce a strong perturbation of lipid-lipid interaction in both phases, especially in L_o_ one, and the changes in lipid-lipid enthalpy have been identified as the main driving force for sorting of the peptides to the L_d_ phase [100]. Another interesting case dealing with phase separation effects is represented by Daptomycin (DAP), a lipopeptide clinically used in treatment of skin infection caused by Gram-positive bacteria, which acts in the co-presence of negatively charged headgroups as phosphatidylglycerol (PG) and calcium ions following specific stoichiometric ratio [101,102,103]. Strong modifications of lipid thermodynamics, along with lateral reorganization of phase-separated micro domains, were observed revealing a preferential interaction of DAP with PG headgroups regardless of their phase, be it gel or liquid disordered phase, and a deeply entering of lipid bilayer in correspondence of such PG moieties [99]. Moreover, the nanomechanical effects observed are strictly dependent on the thermodynamic phase as DAP seems to simultaneously induce domains stiffening when PG is in the fluid phase and softening when it is in the gel phase (Figure 3b,c). 

### 3.2. Effects of Cholesterol-Tuned Membrane Fluidity

Membrane fluidity is another key feature that contributes to shaping the intrinsic complexity of the cell membrane organization. The variable degree of membrane fluidity plays decisive roles under normal and pathological conditions by providing the appropriate environment for the functioning of biomolecules residing within or associated with the membrane bilayer, such as metabolic enzymes, membrane-bound transporters and ion channels. Even in previously introduced phase-separated membranes, the discontinuity in membrane fluidity resulting from lateral lipid segregation is crucial for various cellular processes including signalling and membrane trafficking [104,105]. Numerous physical (temperature, surface charge) and chemical (lipid composition, phospholipid unsaturation, pH) parameters modulate the fluidity properties of membrane bilayers. Cholesterol is arguably the major component of eukaryotic cells that preserves and regulates the functional fluidity of cell membranes over a wide range of temperatures [106]. Mammalian cell membranes account for ~40 to 90% of total cellular cholesterol [107], which in turn constitutes ~20–50 mol % of all membrane lipids depending on cell type and species [108,109]. Cholesterol also contributes to maintaining membrane integrity and modulating lateral phase separation resulting from preferential lipid-lipid interactions [106]. Gaining mechanistic insights into how cholesterol-tuned membrane properties drive encounters with exogenous entities, be they drugs, peptides or nanoparticles, is essential to understand and control the nano-bio interactions that occur at the cellular interface. Membrane fluidity, in particular, is crucial in regulating the permeability of cell membranes to therapeutic molecules and other functional substances for controlled delivery applications.

**Figure 4:**
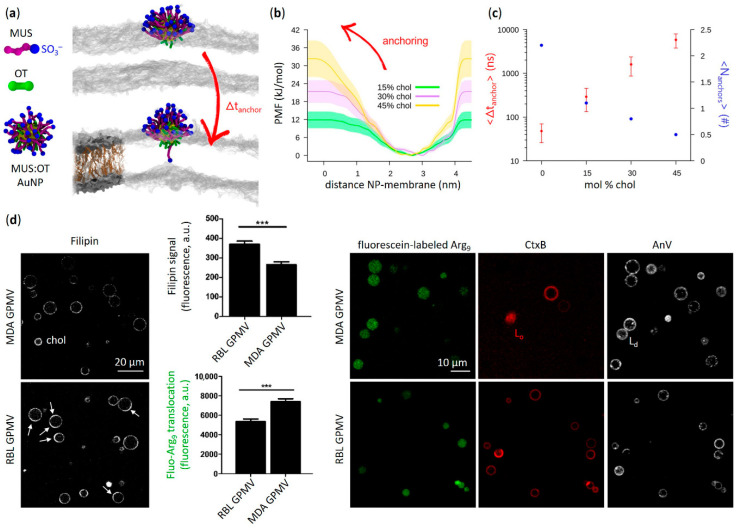
Translocation of amphiphilic AuNPs and amphiphilic peptides is favoured into lipid membranes with lower cholesterol content. (**a**) Left: coarse-grained structure of hydrophilic (MUS) and hydrophobic (OT) ligands and an amphiphilic MUS:OT AuNP in water (2 nm core size; water not shown). Right: simulation snapshots showing the ligand anchoring typical of these AuNPs (see Figure 1b). The NP goes from the hydrophobic contact state (top) to the anchored state (bottom) in which one MUS charged terminal is in contact with the lipid heads (transparent gray) of the distal leaflet. Cholesterol molecules—intercalated between the apolar tails of membrane phospholipids (DOPC)—are shown in tan in the membrane detail on the bottom left. (**b**) Anchoring free energy barriers calculated with well-tempered metadynamics simulations at different membrane cholesterol concentrations. (**c**) Average anchoring time (Δt_anchor_) and average number of anchored ligands after 1 μs (N_anchors_) obtained from unbiased MD simulations as a function of membrane cholesterol content. (**d**) Translocation of the Arg-rich CPP nona-arginine (Arg_9_)—labeled with fluorescein (green)—into GPMVs derived from MDA-MB-231 (MDA GPMV) and RBL-2H3 (RBL GPMV) cells. Left images: GPMVs labeled with filipin (pseudo-colored as white) to bind membrane cholesterol and enable its visualization. Right images: GPMVs labeled with Alexa Fluor 555-conjugated cholera toxin B subunit (CtxB, red) and Alexa Fluor 647-conjugated annexin V (AnV, pseudocolored as white) to visualize, respectively, the L_o_ membrane domains and phosphatidylserine contained in the outer leaflet of the limiting membrane of both vesicle types. Quantification of the filipin signal shows that MDA GPMVs contain approximately 30% less membrane cholesterol than RBL GPMVs; in addition, RBL GPMVs show several large cholesterol-enriched subdomains (white arrows) that are rarer in MDA GPMVs. Overall, Arg_9_ translocation is significantly reduced in vesicles characterized by higher membrane cholesterol content and more cholesterol-rich membrane microdomains. *** *p*-value < 0.0001 and 0.0005 for filipin signal and fluo-Arg_9_ translocation, respectively. (**a**–**c**) contain images by Canepa et al. [45] reprinted with minor modifications—Copyright © 2021 The Authors, published by American Chemical Society. (**d**) is reproduced and adapted with permission from Lorents et al. [111]—Copyright © 2018 American Chemical Society.

Only recently, biologically relevant concentrations of membrane cholesterol have been shown to hinder the molecular mechanism for passive translocation of amphiphilic MUS:OT AuNPs across fluid bilayers (Figure 4a–c) [45]. From a molecular perspective, the stability of passive NP incorporation into the membrane bilayer is highly dependent on the occurrence of lipid fluctuations that pave the way for ligand translocation. The ordering effects of cholesterol on lipid chains reduce lipid dynamics [110], leading to a dramatic decrease in spontaneous NP permeation. This is in agreement with previous observations showing a preferential binding of amphiphilic MUS:OT AuNPs with the fluid phase of lipid membranes and exclusion from highly ordered gel phase domains [44,47].

A similar behavior to that found for MUS:OT AuNPs is observed when analyzing the effect of cholesterol on the membrane uptake of amphipathic peptides endowed with surface conformational flexibility. This is particularly true when considering CPPs enriched in Arg [112], one of the main amino acids showing snorkeling properties [111,113,114,115,116]. As illustrated in Figure 1c, in a manner analogous to thiol ligands of AuNPs, Arg residues are able to stably incorporate within the lipid bilayer by accommodating the uncharged moiety of their flexible side chains into the hydrophobic core of the membrane, while localizing the hydrophilic terminal charges at the lipid head region [14,15,117]. This interaction is thought to enable direct cell membrane penetration of Arg-rich CPPs, a process that has been shown to be stimulated by several physicochemical factors occurring at the membrane interface (e.g., surface concentration of peptides, interplay of membrane counterions, modulation in peptide hydrophobicity and number of Arg residues) [118,119,120]. On the contrary, the presence of membrane cholesterol has been reported to limit the spontaneous permeation of Arg-rich CPPs through lipid membranes (Figure 4d). This is the case, for example, of nona-arginine (Arg_9_) derivatives interacting with lipid bilayers composed of 1,2-dioleoyl-sn-glycero-3-phospho-(1′-rac-glycerol) (DOPG), 1,2-dioleoyl-sn-glycero-3-phosphocholine (DOPC) and cholesterol. Despite polyarginines strongly adsorbed on PG/PC bilayers containing cholesterol [113], their membrane translocation efficiency was drastically reduced [113,114]. In a more biomimetic study by Lorents and co-workers [111], the uptake of Arg-rich amphiphilic CPPs such as Arg_9_ and Tat-peptide (pTat) into giant plasma membrane vesicles (GPMVs) derived from different cell lines was also favoured by decreasing the cholesterol content of the membrane. Previous results by Pae et al. [115] reinforce the experimental evidence that higher cholesterol content severely interferes with the interaction between GPMVs and CPPs containing Arg residues. A high mole fraction of membrane cholesterol has also been shown to suppress the uptake of other amphipathic CPPs such as transportan 10 (TP10) across vesicle bilayers comprising a DOPG/DOPC mixture [117]. Interestingly, the direct translocation of amphipathic CPPs such as transportans and analogues into biomimetic systems was observed to be more strongly inhibited by cholesterol-induced bilayer rigidification with respect to polyarginine Arg_9_ or pTat peptides [115]. Other Arg-based CPPs of prime importance such as penetratin peptides have been reported to exhibit a significant decrease in vesicle uptake efficiency upon cholesterol incorporation into the lipid bilayer [116]. Solid evidence for the impact of cholesterol in inhibiting the entry of membrane-permeable CPPs into cells has also been provided. Key studies by Futaki et al. [118,121] and Watkins et al. [122], for example, clearly show that the conditions for direct translocation of Arg-rich CPPs across living cell membranes are significantly promoted by the loosening of lipid packing induced by cholesterol depletion (e.g., via treatment with methyl-β-cyclodextrin).

As pointed out above, strong similarities in the mechanisms of interaction of lipid membranes with amphiphilic AuNPs and amphiphilic peptides can also be found when membrane-active AMPs, which share analogous physicochemical properties to CPPs, are considered [123]. Even in this context, certain amino acid sequences, such as Arg, Lys and histidine residues, contribute to establishing favourable hydrophobic and electrostatic interactions with the different regions of the lipid bilayer [124], assisting the peptide translocation across the membrane [125,126]. When looking at the combination of cholesterol and zwitterionic PC phospholipids in biomimetic systems, numerous examples reveal a dramatic reduction in the membrane-binding properties of Arg-containing amphipathic AMPs [127]. This is the case, for instance, of honeybee melittin (one of the most studied antimicrobial peptides) [128], temporin L derived from the frog Rana temporaria [129] and Arg-rich protegrin-1 isolated from porcine leukocytes [130,131]. Remarkably, in homogeneous PC bilayers with a high cholesterol content, the spontaneous membrane incorporation of melittin has been reported to be strongly delayed compared to phase-separated lipid systems with the same amount of cholesterol [132]. This result confirms that lateral phase separation is an important factor driving the membrane activity of peptides exhibiting hydrophilic and hydrophobic properties, in agreement with previous studies involving amphiphilic thiol-protected AuNPs [44,77,82]. Cholesterol-attenuated peptide insertion into zwitterionic or anionic PG-containing membranes has also been demonstrated for many known amphipathic AMPs that do not include Arg residues, including gramicidin S [133], maculatin 1.1 [134], pleurocidin [135] and the LL-37 peptide belonging to the cathelicidin family [129]. Based on this general behavior, studies conducted in recent decades increasingly attribute a protective role to cholesterol in eukaryotic membranes against damage caused by host AMPs [72,73,74]. Indeed, depending on the membrane content in each cell type, cholesterol acts as an effective molecular regulator of antimicrobial peptide-membrane interactions, making the membrane more resistant to structural transformations induced by embedding AMPs.

### 3.3. Aggregation Effects on the Membrane Surface

In the previous paragraphs, we observed how exogenous entities with amphiphilic properties such as synthetic NPs or peptides can passively and stably penetrate the membrane bilayer. Moreover, we remarked that the biological membrane has a multicomponent nature, meaning that many different macromolecules, mainly proteins, are structurally embedded in the lipid bilayer frame. We can refer to all bilayer-embedded entities as membrane inclusions, independently from their biological or synthetic nature. The interactions in these systems can then be categorized into inclusion-lipid interactions, lipid-lipid interactions, and inclusion-inclusion interactions, all of which can be mutually influenced, as well as influenced by the interactions with the solvent. We have already addressed the case of the inclusion-lipid interaction: the process of membrane penetration of amphiphilic AuNPs and amphiphilic peptides, and the impact of membrane cholesterol in hindering this event. Moreover, the perturbation of membrane lateral phase separation represents a clear case of the impact of membrane inclusions on lipid-lipid interactions. However, if we consider more than a single inclusion, we must also consider direct and indirect inclusion-inclusion interactions, the combination of which can lead to the aggregation of membrane inclusions. 

Membrane proteins offer several instructive examples of how the aggregation of biological membrane inclusions is crucial in allowing many of their functions to be carried out [136,137]. Several membrane receptors, such as tyrosine kinase and G-protein coupled receptors, can in fact only be activated after oligomerization [138,139]. In other cases, the cell takes advantage of the aggregation of membrane proteins to stabilize functional structures of the lipid bilayer: the dimerization of caveolin proteins, for instance, allows the formation of membrane folds called caveolae [140]. Proteins with a ‘Bin/Amphiphysin/Rvs’ (BAR) domain also work by scaffolding and assembling [141,142,143]. Aggregation is also part of the functioning mechanisms of smaller peptide molecules, as in the case of AMPs that are able to disrupt the membrane by creating pores [144,145]. According to both the barrel-stave and toroidal model, the mechanism of pore formation requires peptide oligomerization [146,147,148]. Protein and peptide aggregation is also involved in pathological processes, such as amyloid formation [149]. Given the similarity between the behavior of amphiphilic AuNPs and numerous examples of amphiphilic peptide families, we expect the aggregation of membrane-embedded NPs to affect membrane structure in a similar way. Ideally, the NP aggregation tendency could be controlled to prevent their potential toxicity [150] or exploited for targeted biomedical applications [151]. 

**Figure 5:**
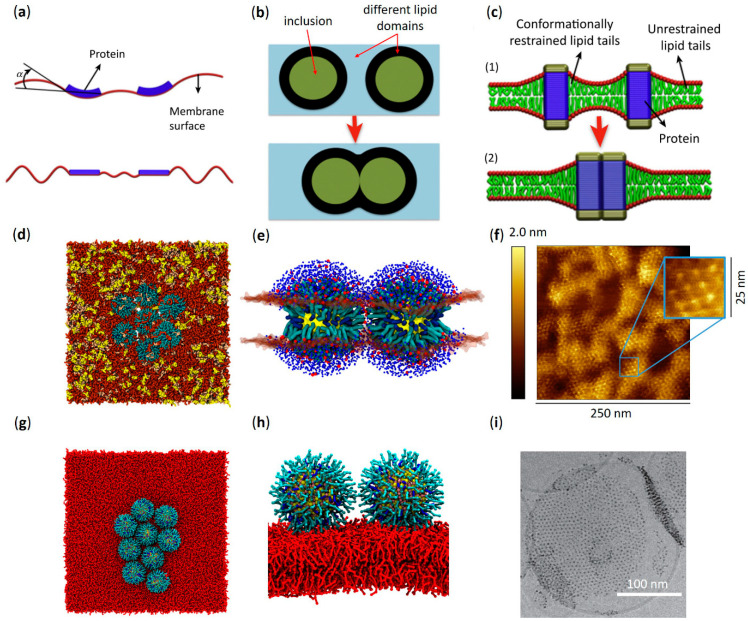
The tendency for membrane aggregation is shared by amphiphilic AuNPs and amphiphilic peptides. (**a**) Top, sketch of two curvature inducing membrane inclusions; due to the elastic energy of the membrane, an effective interacting potential can arise and, depending on the system, the interaction can be attractive and thus lead to aggregation. Bottom, system in which the inclusions suppress the natural fluctuations of the membrane; aggregation can minimize this region. (**b**) Aggregation induced by lipid depletion. (**c**) Aggregation induced by capillary forces. In configuration (1), there are two regions of modified lipid density around the inclusion; dimerization allows to minimize their total area, as shown in configuration (2). (**d**) Ordered aggregate of MUS:OT NPs adsorbed on a DOPC membrane. The snapshot is taken from unbiased MD simulations (Martini CG). Nanoparticles are represented with yellow beads (Au), pink beads (S), cyan (MUS ligands) and blue (OT ligands); membrane lipids are represented with red beads. (**e**) Dimer of adsorbed NPs on DOPC membrane from unbiased MD simulations (Martini CG). The extended ligand configuration can be observed. Representation as in (**d**). (**f**) Cryo-EM image of MUS:OT aggregation on the surface of a DOPC liposome. The NP-NP is compatible with the extended ligand configuration. (**g**) Ordered aggregate of MUS:OT NPs embedded in a model neuronal plasma membrane. The snapshot is taken from unbiased MD simulations (Martini CG). The NP are represented with a yellow core, blue OT ligands and cyan MUS ligands. The membrane is represented with red DliPC lipids, light pink sphingomyelin, yellow ganglioside and grey cholesterol. (**h**) Dimer of MUS:OT NPs embedded in a model neuronal plasma membrane. The deformation of the ligand shell and the presence of the stabilizing layer of ions (red beads) can be observed. The membrane headgroups are shown as semi-transparent surface, lipid tails are not shown for clarity. (**i**) Supramolecular lattice formed by M1 bilayer-embedded MUS:OT NPS, imaged by AFM. The digital zoom of the area with blue contour shows the lattice order at higher magnification. (**a**–**c**) adapted with permission from Johannes et al. [152] Copyright © 2022 Elsevier B.V. All rights reserved. (**d**–**f**) adapted from Lavagna et al. [173] with permission from the Royal Society of Chemistry. (**g**–**i**) adapted from Canepa et al. [44] under a CC BY-NC 3.0 license with permission from the Royal Society of Chemistry.

The driving force for membrane inclusion aggregation can vary significantly at different length scales. For instance, inclusions of tens or hundreds of nanometers typically interact thanks to motor-driven systems, in which the motor is composed of actin complexes in various configurations [152,153,154,155]. In the case of smaller inclusions, the dominating driving force at medium range is usually the minimization of the membrane elastic energy. Indeed, for distances larger than the typical bilayer thickness (4–5 nm), the membrane can be seen as a thin, flexible sheet [142,152,156], for which deformations come at an energy cost given by the Helfrich Hamiltonian [157]. In such a scheme, when inclusions are inserted, they modify the local curvature of the membrane. In response, the membrane changes the distribution of the inclusions to minimize the overall elastic energy (Figure 5a). The resulting effective inclusion-inclusion interactions can be quite complex when considered at the collective level [158,159,160]. Shallow membrane deformations often induce repulsion [152,161,162], but in some instances, the resulting interaction can be attractive, leading to aggregation [163,164]. When considering very short length scales (in the order of 1 nm), the effects of the finite size of the lipids, with their partial order and ability to bend, rotate and tilt, cannot be neglected. In multicomponent lipid bilayers, the inclusions can induce domain formation in their surrounding area; in this case, aggregation is driven by the minimization of the interfacial tension between the separated domains (capillary forces, Figure 5c) [165,166,167,168]. Inclusions can also create a region of lipid depletion: this happens if the conformational freedom of the lipids is somewhat constrained. For instance, the height mismatch between the hydrophobic surface of the inclusion and the lipid tails can induce lipid depletion (Figure 5b). As for capillary forces, aggregation is favored because it minimizes the extent of the depleted region [169,170,171,172]. 

The interaction mechanisms described above were originally characterized for protein and peptide inclusions but may remain valid for protein-mimicking objects such as amphiphilic AuNPs. Mechanisms involving subtle perturbations of membrane lipids are particularly suited to be studied using molecular models and MD simulations. Simulations from Chan et al. [174] proved that membrane aggregation of sub-2-nm-AuNPs coated with hydrophobic thiol ligands depends on the strength of ligand-lipid interactions; this in silico result closely reproduces the experimental observations reported by Rasch et al. using cryogenic transmission electron microscopy (cryo-TEM) [175]. Interestingly, recent coarse-grained MD results from Angelikopoulos et al. [176] highlighted the complexity of the aggregation mechanisms of amphiphilic MUS:OT AuNPs. In their study, AuNPs with a core diameter of 3 nm and a 1:1 MUS:OT surface composition self-assemble in a 1,2-dipalmitoyl-sn-glycero-3-phosphocholine (DPPC) membrane containing cholesterol. They explain the aggregation showing that NPs induce a region of cholesterol depletion, in which lipids form a liquid disordered phase, rather than a liquid ordered bulk phase. Thus, NP aggregation minimizes liquid ordered-liquid disordered interface. Moreover, they observe that the shape of the aggregates is linear, which cannot be explained only with the minimization of the liquid ordered-liquid disordered interface. The linear geometry depends on the reshaping ability of the ligand shell: both the NP-NP and the NP-lipid interfaces are stabilized by the MUS ligand terminals, which are in a finite number. Thus, each embedded MUS:OT AuNP can form only a finite number of NP-NP bonds before their membrane embedding becomes unstable. It is worth noting that this effect is dependent on the surface density of the charged terminals of the thiol ligands, which strongly depends on the NP core size and ligand monolayer composition. These properties were not explored in this work, implying that the generality of the process is not guaranteed. However, linear aggregation is not even peculiar to soft-shell NPs: it can also be explained by membrane curvature forces, as recently demonstrated in the case of rigid hydrophobic NPs [177]. Still focusing on the case of amphiphilic MUS:OT AuNPs, an interesting effect of the NP core size on bilayer aggregation was observed in silico in a recent study by Canepa et al. [44], where the NPs were free to diffuse in a quaternary mixture of PC, SM, cholesterol, and ganglioside GM1 mimicking the neuronal plasma membrane. In this study, embedded NPs of two different core sizes (2 and 4 nm) were used, both with a MUS:OT ligand shell in a 2:1 ratio (Figure 1a). While embedded 2 nm-AuNPs did not show any tendency to aggregate, 4 nm-AuNPs formed well ordered, hexagonal lattices (Figure 5d). The authors concluded that the transition from the non-aggregation to the aggregation regime happens somewhere between the 2 and 4 nm size range. Again, NP-NP dimerization was mediated by the charged terminals of MUS ligands (Figure 5e). The difference in aggregation geometry compared to the results of Angelikopoulos et al. [176] can be explained by the different size of the AuNPs and the different composition of the surface ligand shell: a larger NP size and a higher MUS concentration may in fact result in a higher density of charged ligands, allowing each NP to form more NP-NP bonds. Furthermore, Canepa and co-workers [44] note a possible correlation between the degree of NP aggregation and the degree of NP embedding within the membrane. Their computational results are corroborated by experimental AFM investigations performed on lipid bilayers of similar composition. The AFM images clearly confirm the formation of bilayer-embedded NP aggregates and their highly ordered geometry. Remarkably, the experimental NP-NP spacing within the aggregates is coherent with the extended ligands configuration of the MD simulations [44], as shown in Figure 5f. 

A recent work from Lavagna et al. [173] exploits coarse-grained MD simulations to investigate the aggregation driving forces of amphiphilic MUS:OT AuNPs, focusing on the effects of different NP-membrane penetration states. The NP models employed in this study have a 4 nm core diameter, and the membrane is composed of a single lipid (DOPC) in its liquid disordered phase. The authors observe the formation of ordered planar aggregates at all the investigated penetration stages (adsorbed, semi-snorkeled, and fully snorkeled) (Figure 1b) while highlighting different driving forces, as detailed below. Adsorbed planar aggregates (Figure 5g,h) on the surface of the membrane are due to ion-bridging: a layer of positive counterions glues the negatively charged NP interfaces. This ion-mediated interaction is a short-range phenomenon that happens in the water phase. It is a peculiar, non-DLVO driving force present also in the absence of membranes, as shown by Petretto et al. with atomistic simulations [178] and experimental images (Figure 5i). The fact that ion-bridged aggregates can be found in water implies that the aggregates adsorbed on the membrane could come from two different pathways: from the lateral diffusion of single adsorbed NPs or from the flattening of a NP cluster formed in the water phase. For semi-snorkeled NPs, ordered aggregates still form, but the aggregation kinetics are somewhat slower. Indeed, while the aggregation is again stabilized at a short range by ion-bridging, the strong membrane curvature that the NPs induce on the bilayer generates a free energy barrier in the dimerization process. To form a dimer, the deformations induced by two NPs must become a large one, with intermediate configurations with a high curvature point, implying a high energy cost. Thus, in this example, the elastic energy of the membrane contributes to shaping the attractive inclusion-inclusion interactions. Finally, for fully snorkeled NPs, a longer-range interaction is observed and explained by measuring a lipid tail depletion aura around NPs. When two lipid depletion auras come in contact, the particles start attracting each other. Then, once again, ion briding can stabilize the aggregation at short range. Numerous cryo-TEM images confirm the presence of MUS:OT AuNP aggregates in different penetration configurations on the membrane of DOPC liposomes [47,179]. As in the work of Canepa et al. previously discussed [44], also in this context the measured NP-NP distance in ordered aggregates is compatible with the extended ligand configuration observed in simulations [173]. Based on all these theoretical and experimental observations, it is important to remark that the reshaping of the NP ligand shell allows these amphiphilic AuNPs to display a wide variety of behaviors in terms of formation of different stable NP-membrane configurations. These could be finely tuned through minimal changes not only in the composition of the NP ligand shell, but also in the size of the gold core.

## 4. Conclusions

A large number of functionalization strategies have been proposed to decorate the surface of gold nanoparticles resulting in an amphiphilic behavior for targeted biological applications [180,181,182,183,184]. When interacting with lipid membranes, amphiphilic AuNPs protected by mixed thiol monolayers share remarkable similarities with well-known functional peptides endowed with amphipathic properties and biomedical potential. These include, for example, cell-penetrating peptides and antimicrobial peptides that have emerged as interesting categories of therapeutic agents. Analogous to many examples in these peptide families, amphiphilic AuNPs are able to spontaneously insert into the lipid bilayer through a penetration mechanism that relies on a ligand snorkeling process, which results in the translocation of flexible ω-charged ligands across the hydrocarbon moiety of the membrane bilayer from one polar interface to the opposite one. Furthermore, amphiphilic AuNPs are able to undergo aggregation in the membrane, in parallel to oligomerization processes exhibited by antimicrobial peptides and, in general, to functional or pathologic aggregation of peptides and proteins. Both AuNPs and peptides also preferentially interact with the L_d_ phase of the bilayer, being excluded from highly ordered gel phase domains, and can induce lateral reorganization of phase-separated lipid systems. Finally, their uptake in the bilayer is strictly modulated by the membrane cholesterol content, which is known to play a key role in the regulation of membrane rigidity. 

The strong similarities between nanoparticle membrane and peptide-membrane interactions suggest that the passive incorporation into cell membranes of amphiphilic agents with biomedical potential could target the membrane fluid phase and could be regulated, *in vitro*, by modulating the cholesterol-related membrane fluidity. From an opposite perspective, the effects of membrane cholesterol and phase separation in governing the dynamic processes of eukaryotic cell membranes should be taken into high consideration when interpreting and/or bioengineering the direct and selective uptake of amphiphilic AuNPs or amphiphilic peptides into cells *in vivo*. This certainly has a strong impact on the biological activity of both nanoparticles and peptides; however, the molecular details underlying the mechanism of action between nanoparticles or peptides and the membrane environment have yet to be fully elucidated.

Collectively, the evidence reported in this review indicates that amphiphilic AuNPs protected by thiol ligands may provide an excellent model for elucidating the mechanistic aspects of the interaction between functional peptides and the lipid bilayer of cell membranes. In addition, it reveals the extreme biological versatility of amphiphilic AuNPs, which constitute a very powerful tool not only for simulating the interaction with the membrane but also in the control and regulation of this interaction by tailoring the size of the NP core and the design of the core-shell assembly [40,47], thus allowing a synthetic biology approach to gain insight into biologically relevant membrane processes. In this scenario, a very interesting example is the recently demonstrated ability of amphiphilic thiol-protected AuNPs to promote Ca^2+^-triggered membrane fusion in a manner similar to much more complex endogenous protein structures [185]. Overall, this biomimetic approach could open the way for more systematic studies at the nanoscale that could ultimately lead to the development of engineered NPs with rationally designed biosynthetic activity for next-generation nanomedicine-based technologies. Among others, these can include novel agents for cancer therapy and treatment of bacterial infections, as well as finely tuned vehicles for intracellular delivery.

## References

[1] Apostolopoulos V., Bojarska J., Chai T.-T., Elnagdy S., Kaczmarek K., Matsoukas J., New R., Parang K., Lopez O.P., Parhiz H. (2021). A Global Review on Short Peptides: Frontiers and Perspectives. Molecules.

[2] Ko J., Auyeung K. (2014). Identification of Functional Peptides from Natural and Synthetic Products on Their Anticancer Activities by Tumor Targeting. CMC.

[3] Wang X.-Y., Wang Y.-H., Song Z., Hu X.-Y., Wei J.-P., Zhang J., Wang H.-S. (2021). Recent Progress in Functional Peptides Designed for Tumor-Targeted Imaging and Therapy. J. Mater. Chem. C.

[4] Rong L., Qin S.-Y., Zhang C., Cheng Y.-J., Feng J., Wang S.-B., Zhang X.-Z. (2018). Biomedical Applications of Functional Peptides in Nano-Systems. Mater. Today Chem..

[5] Oyston P.C.F., Fox M.A., Richards S.J., Clark G.C. (2009). Novel Peptide Therapeutics for Treatment of Infections. J. Med. Microbiol..

[6] Hoffmann A.R., Guha S., Wu E., Ghimire J., Wang Y., He J., Garry R.F., Wimley W.C. (2020). Broad-Spectrum Antiviral Entry Inhibition by Interfacially Active Peptides. J. Virol..

[7] Sánchez A., Vázquez A. (2017). Bioactive Peptides: A Review. Food Qual. Saf..

[8] Macedo M.W.F.S., da Cunha N.B., Carneiro J.A., da Costa R.A., de Alencar S.A., Cardoso M.H., Franco O.L., Dias S.C. (2021). Marine Organisms as a Rich Source of Biologically Active Peptides. Front. Mar. Sci..

[9] Kalafatovic D., Giralt E. (2017). Cell-Penetrating Peptides: Design Strategies beyond Primary Structure and Amphipathicity. Molecules.

[10] Luzio J.P., Pryor P.R., Bright N.A. (2007). Lysosomes: Fusion and Function. Nat. Rev. Mol. Cell Biol..

[11] LeCher J.C., Nowak S.J., McMurry J.L. (2017). Breaking in and Busting out: Cell-Penetrating Peptides and the Endosomal Escape Problem. Biomol. Concepts.

[12] Strandberg E., Killian J.A. (2003). Snorkeling of Lysine Side Chains in Transmembrane Helices: How Easy Can It Get?. FEBS Lett..

[13] Schow E.V., Freites J.A., Myint P.C., Bernsel A., von Heijne G., White S.H., Tobias D.J. (2011). Arginine in Membranes: The Connection Between Molecular Dynamics Simulations and Translocon-Mediated Insertion Experiments. J. Membr. Biol..

[14] Keskin A., Akdoğan E., Dunn C.D. (2017). Evidence for Amino Acid Snorkeling from a High-Resolution, in Vivo Analysis of Fis1 Tail-Anchor Insertion at the Mitochondrial Outer Membrane. Genetics.

[15] Saha S., Ghosh A., Tiwari N., Kumar A., Kumar A., Goswami C. (2017). Preferential Selection of Arginine at the Lipid-Water-Interface of TRPV1 during Vertebrate Evolution Correlates with Its Snorkeling Behaviour and Cholesterol Interaction. Sci. Rep..

[16] Jafari M., Mehrnejad F., Doustdar F. (2017). Insight into the Interactions, Residue Snorkeling, and Membrane Disordering Potency of a Single Antimicrobial Peptide into Different Lipid Bilayers. PLoS ONE.

[17] Mishra V.K., Palgunachari M.N., Segrest J.P., Anantharamaiah G.M. (1994). Interactions of Synthetic Peptide Analogs of the Class A Amphipathic Helix with Lipids. Evidence for the Snorkel Hypothesis. J. Biol Chem.

[18] Shinoda W. (2016). Permeability across Lipid Membranes. Biochim. Et Biophys. Acta (BBA) Biomembr..

[19] Tejwani R.W., Davis M.E., Anderson B.D., Stouch T.R. (2011). An Atomic and Molecular View of the Depth Dependence of the Free Energies of Solute Transfer from Water into Lipid Bilayers. Mol. Pharm..

[20] Filipe H.A.L., Cardoso R.M.S., Loura L.M.S., Moreno M.J., Chattopadhyay A. (2017). Interaction of Amphiphilic Molecules with Lipid Bilayers: Ki-netics of Insertion, Desorption and Translocation. Membrane Organization and Dynamics.

[21] Liu X., Testa B., Fahr A. (2011). Lipophilicity and Its Relationship with Passive Drug Permeation. Pharm. Res..

[22] Zhang R., Qin X., Kong F., Chen P., Pan G. (2019). Improving Cellular Uptake of Therapeutic Entities through Interaction with Components of Cell Membrane. Drug Deliv..

[23] Vorobyov I., Bekker B., Allen T.W. (2010). Electrostatics of Deformable Lipid Membranes. Biophys. J..

[24] MacCallum J.L., Tieleman D.P. (2011). Hydrophobicity Scales: A Thermodynamic Looking Glass into Lipid–Protein Interactions. Trends Biochem. Sci..

[25] Rezai T., Bock J.E., Zhou M.V., Kalyanaraman C., Lokey R.S., Jacobson M.P. (2006). Conformational Flexibility, Internal Hydrogen Bonding, and Passive Membrane Permeability:  Successful in Silico Prediction of the Relative Permeabilities of Cyclic Peptides. J. Am. Chem. Soc..

[26] Galdiero S., Falanga A., Cantisani M., Vitiello M., Morelli G., Galdiero M. (2013). Peptide-Lipid Interactions: Experiments and Applications. IJMS.

[27] Sanderson J.M. (2005). Peptide–Lipid Interactions: Insights and Perspectives. Org. Biomol. Chem..

[28] Yang Y.-S.S., Atukorale P.U., Moynihan K.D., Bekdemir A., Rakhra K., Tang L., Stellacci F., Irvine D.J. (2017). High-Throughput Quantitation of Inorganic Nanoparticle Biodistribution at the Single-Cell Level Using Mass Cytometry. Nat. Commun..

[29] Verma A., Stellacci F. (2010). Effect of Surface Properties on Nanoparticle–Cell Interactions. Small.

[30] Verma A., Uzun O., Hu Y., Hu Y., Han H.-S., Watson N., Chen S., Irvine D.J., Stellacci F. (2008). Surface-Structure-Regulated Cell-Membrane Penetration by Monolayer-Protected Nanoparticles. Nat. Mater..

[31] Jewell C.M., Jung J.-M., Atukorale P.U., Carney R.P., Stellacci F., Irvine D.J. (2011). Oligonucleotide Delivery by Cell-Penetrating “Striped” Nanoparticles. Angew. Chem. Int. Ed..

[32] Carney R.P., Carney T.M., Mueller M., Stellacci F. (2012). Dynamic Cellular Uptake of Mixed-Monolayer Protected Nanoparticles. Biointerphases.

[33] Leduc C., Jung J.-M., Carney R.R., Stellacci F., Lounis B. (2011). Direct Investigation of Intracellular Presence of Gold Nanoparticles *via* Photothermal Heterodyne Imaging. ACS Nano.

[34] Atukorale P.U., Yang Y.-S., Bekdemir A., Carney R.P., Silva P.J., Watson N., Stellacci F., Irvine D.J. (2015). Influence of the Glycocalyx and Plasma Membrane Composition on Amphiphilic Gold Nanoparticle Association with Erythrocytes. Nanoscale.

[35] Sabella S., Carney R.P., Brunetti V., Malvindi M.A., Al-Juffali N., Vecchio G., Janes S.M., Bakr O.M., Cingolani R., Stellacci F. (2014). A General Mechanism for Intracellular Toxicity of Metal-Containing Nanoparticles. Nanoscale.

[36] Dreaden E.C., Alkilany A.M., Huang X., Murphy C.J., El-Sayed M.A. (2012). The Golden Age: Gold Nanoparticles for Biomedicine. Chem. Soc. Rev..

[37] Pengo P., Şologan M., Pasquato L., Guida F., Pacor S., Tossi A., Stellacci F., Marson D., Boccardo S., Pricl S. (2017). Gold Nanoparticles with Patterned Surface Monolayers for Nanomedicine: Current Perspectives. Eur. Biophys. J..

[38] Simonelli F., Bochicchio D., Ferrando R., Rossi G. (2015). Monolayer-Protected Anionic Au Nanoparticles Walk into Lipid Membranes Step by Step. J. Phys. Chem. Lett..

[39] Salassi S., Simonelli F., Bochicchio D., Ferrando R., Rossi G. (2017). Au Nanoparticles in Lipid Bilayers: A Comparison between Atomistic and Coarse-Grained Models. J. Phys. Chem. C.

[40] Van Lehn R.C., Atukorale P.U., Carney R.P., Yang Y.-S., Stellacci F., Irvine D.J., Alexander-Katz A. (2013). Effect of Particle Diameter and Surface Composition on the Spontaneous Fusion of Monolayer-Protected Gold Nanoparticles with Lipid Bilayers. Nano Lett..

[41] Lane J.M.D., Grest G.S. (2010). Spontaneous Asymmetry of Coated Spherical Nanoparticles in Solution and at Liquid-Vapor Interfaces. Phys. Rev. Lett..

[42] Henriques S.T., Melo M.N., Castanho M.A.R.B. (2006). Cell-Penetrating Peptides and Antimicrobial Peptides: How Different Are They?. Biochem. J..

[43] Van Lehn R.C., Ricci M., Silva P.H.J., Andreozzi P., Reguera J., Voïtchovsky K., Stellacci F., Alexander-Katz A. (2014). Lipid Tail Protrusions Mediate the Insertion of Nanoparticles into Model Cell Membranes. Nat. Commun..

[44] Canepa E., Salassi S., de Marco A.L., Lambruschini C., Odino D., Bochicchio D., Canepa F., Canale C., Dante S., Brescia R. (2020). Amphiphilic Gold Nanoparticles Perturb Phase Separation in Multidomain Lipid Membranes. Nanoscale.

[45] Canepa E., Bochicchio D., Gasbarri M., Odino D., Canale C., Ferrando R., Canepa F., Stellacci F., Rossi G., Dante S. (2021). Cholesterol Hinders the Passive Uptake of Amphiphilic Nanoparticles into Fluid Lipid Membranes. J. Phys. Chem. Lett..

[46] Canepa E., Salassi S., Simonelli F., Ferrando R., Rolandi R., Lambruschini C., Canepa F., Dante S., Relini A., Rossi G. (2021). Non-Disruptive Uptake of Anionic and Cationic Gold Nanoparticles in Neutral Zwitterionic Membranes. Sci. Rep..

[47] Atukorale P.U., Guven Z.P., Bekdemir A., Carney R.P., Van Lehn R.C., Yun D.S., Jacob Silva P.H., Demurtas D., Yang Y.-S., Alexander-Katz A. (2018). Structure–Property Relationships of Amphiphilic Nanoparticles That Penetrate or Fuse Lipid Membranes. Bioconjugate Chem..

[48] Carney R.P., Astier Y., Carney T.M., Voïtchovsky K., Jacob Silva P.H., Stellacci F. (2013). Electrical Method to Quantify Nanoparticle Interaction with Lipid Bilayers. ACS Nano.

[49] Yang Y.-S.S., Moynihan K.D., Bekdemir A., Dichwalkar T.M., Noh M.M., Watson N., Melo M., Ingram J., Suh H., Ploegh H. (2019). Targeting Small Molecule Drugs to T Cells with Antibody-Directed Cell-Penetrating Gold Nanoparticles. Biomater. Sci..

[50] Uzun O., Hu Y., Verma A., Chen S., Centrone A., Stellacci F. (2008). Water-Soluble Amphiphilic Gold Nanoparticles with Structured Ligand Shells. Chem. Commun..

[51] Cao Z., Liu L., Hu G., Bian Y., Li H., Wang J., Zhou Y. (2020). Interplay of Hydrophobic and Hydrophilic Interactions in Sequence-Dependent Cell Penetration of Spontaneous Membrane-Translocating Peptides Revealed by Bias-Exchange Metadynamics Simulations. Biochim. Et Biophys. Acta (BBA) Biomembr..

[52] Heikkilä E., Martinez-Seara H., Gurtovenko A.A., Vattulainen I., Akola J. (2014). Atomistic Simulations of Anionic Au144(SR)60 Nanoparticles Interacting with Asymmetric Model Lipid Membranes. Biochim. Et Biophys. Acta (BBA) Biomembr..

[53] Lehn R.C.V., Alexander-Katz A. (2015). Pathway for Insertion of Amphiphilic Nanoparticles into Defect-Free Lipid Bilayers from Atomistic Molecular Dynamics Simulations. Soft Matter.

[54] Gkeka P., Sarkisov L., Angelikopoulos P. (2013). Homogeneous Hydrophobic–Hydrophilic Surface Patterns Enhance Permeation of Nanoparticles through Lipid Membranes. J. Phys. Chem. Lett..

[55] Lehn R.C.V., Alexander-Katz A. (2013). Free Energy Change for Insertion of Charged, Monolayer-Protected Nanoparticles into Lipid Bilayers. Soft Matter.

[56] Li Y., Li X., Li Z., Gao H. (2012). Surface-Structure-Regulated Penetration of Nanoparticles across a Cell Membrane. Nanoscale.

[57] Lehn R.C.V., Alexander-Katz A. (2011). Penetration of Lipid Bilayers by Nanoparticles with Environmentally-Responsive Surfaces: Simulations and Theory. Soft Matter.

[58] Rossi G., Monticelli L. (2016). Gold Nanoparticles in Model Biological Membranes: A Computational Perspective. Biochim. Et Biophys. Acta (BBA) Biomembr..

[59] Lin J., Zhang H., Chen Z., Zheng Y. (2010). Penetration of Lipid Membranes by Gold Nanoparticles: Insights into Cellular Uptake, Cytotoxicity, and Their Relationship. ACS Nano.

[60] Lin J., Alexander-Katz A. (2013). Cell Membranes Open “Doors” for Cationic Nanoparticles/Biomolecules: Insights into Uptake Kinetics. ACS Nano.

[61] Heikkilä E., Martinez-Seara H., Gurtovenko A.A., Javanainen M., Häkkinen H., Vattulainen I., Akola J. (2014). Cationic Au Nanoparticle Binding with Plasma Membrane-like Lipid Bilayers: Potential Mechanism for Spontaneous Permeation to Cells Revealed by Atomistic Simulations. J. Phys. Chem. C.

[62] Salassi S., Canepa E., Ferrando R., Rossi G. (2019). Anionic Nanoparticle-Lipid Membrane Interactions: The Protonation of Anionic Ligands at the Membrane Surface Reduces Membrane Disruption. RSC Adv..

[63] Reid L.M., Verma C.S., Essex J.W. (2019). The Role of Molecular Simulations in Understanding the Mechanisms of Cell-Penetrating Peptides. Drug Discov. Today.

[64] Fuselier T., Wimley W.C. (2017). Spontaneous Membrane Translocating Peptides: The Role of Leucine-Arginine Consensus Motifs. Biophys. J..

[65] Zorko M., Langel Ü. (2005). Cell-Penetrating Peptides: Mechanism and Kinetics of Cargo Delivery. Adv. Drug Deliv. Rev..

[66] Dorairaj S., Allen T.W. (2007). On the Thermodynamic Stability of a Charged Arginine Side Chain in a Transmembrane Helix. Proc. Natl. Acad. Sci. USA.

[67] Almeida P.F., Pokorny A. (2009). Mechanisms of Antimicrobial, Cytolytic, and Cell-Penetrating Peptides: From Kinetics to Thermodynamics. Biochemistry.

[68] Hu Y., Patel S. (2016). Thermodynamics of Cell-Penetrating HIV1 TAT Peptide Insertion into PC/PS/CHOL Model Bilayers through Transmembrane Pores: The Roles of Cholesterol and Anionic Lipids. Soft Matter.

[69] Herce H.D., Garcia A.E. (2007). Molecular Dynamics Simulations Suggest a Mechanism for Translocation of the HIV-1 TAT Peptide across Lipid Membranes. Proc. Natl. Acad. Sci. USA.

[70] Cruz J., Mihailescu M., Wiedman G., Herman K., Searson P.C., Wimley W.C., Hristova K. (2013). A Membrane-Translocating Peptide Penetrates into Bilayers without Significant Bilayer Perturbations. Biophys. J..

[71] Macchi S., Signore G., Boccardi C., Di Rienzo C., Beltram F., Cardarelli F. (2015). Spontaneous Membrane-Translocating Peptides: Influence of Peptide Self-Aggregation and Cargo Polarity. Sci. Rep..

[72] Hu Y., Patel S. (2015). Structural and Thermodynamic Insight into Spontaneous Membrane-Translocating Peptides Across Model PC/PG Lipid Bilayers. J. Membr. Biol..

[73] Ulmschneider J.P. (2017). Charged Antimicrobial Peptides Can Translocate across Membranes without Forming Channel-like Pores. Biophys. J..

[74] Melby E.S., Mensch A.C., Lohse S.E., Hu D., Orr G., Murphy C.J., Hamers R.J., Pedersen J.A. (2016). Formation of Supported Lipid Bilayers Containing Phase-Segregated Domains and Their Interaction with Gold Nanoparticles. Environ. Sci. Nano.

[75] Sheikh K.H., Jarvis S.P. (2011). Crystalline Hydration Structure at the Membrane–Fluid Interface of Model Lipid Rafts Indicates a Highly Reactive Boundary Region. J. Am. Chem. Soc..

[76] Andersen O.S., Koeppe R.E. (2007). Bilayer Thickness and Membrane Protein Function: An Energetic Perspective. Annu. Rev. Biophys. Biomol. Struct..

[77] Cordeiro R.M. (2018). Molecular Structure and Permeability at the Interface between Phase-Separated Membrane Domains. J. Phys. Chem. B.

[78] Kirsch S.A., Böckmann R.A. (2019). Coupling of Membrane Nanodomain Formation and Enhanced Electroporation near Phase Transition. Biophys. J..

[79] Sheavly J.K., Pedersen J.A., Van Lehn R.C. (2019). Curvature-Driven Adsorption of Cationic Nanoparticles to Phase Boundaries in Multicomponent Lipid Bilayers. Nanoscale.

[80] Balleza D., Mescola A., Marín–Medina N., Ragazzini G., Pieruccini M., Facci P., Alessandrini A. (2019). Complex Phase Behavior of GUVs Containing Different Sphingomyelins. Biophys. J..

[81] Säälik P., Niinep A., Pae J., Hansen M., Lubenets D., Langel Ü., Pooga M. (2011). Penetration without Cells: Membrane Translocation of Cell-Penetrating Peptides in the Model Giant Plasma Membrane Vesicles. J. Control. Release.

[82] Trabulo S., Cardoso A.L., Mano M., De Lima M.C.P. (2010). Cell-Penetrating Peptides—Mechanisms of Cellular Uptake and Generation of Delivery Systems. Pharmaceuticals.

[83] Morris M.C., Deshayes S., Heitz F., Divita G. (2008). Cell-Penetrating Peptides: From Molecular Mechanisms to Therapeutics. Biol. Cell.

[84] Nguyen L.T., Haney E.F., Vogel H.J. (2011). The Expanding Scope of Antimicrobial Peptide Structures and Their Modes of Action. Trends Biotechnol..

[85] Wimley W.C. (2010). Describing the Mechanism of Antimicrobial Peptide Action with the Interfacial Activity Model. ACS Chem. Biol..

[86] Wimley W.C., Hristova K. (2011). Antimicrobial Peptides: Successes, Challenges and Unanswered Questions. J. Membr. Biol..

[87] Bahar A., Ren D. (2013). Antimicrobial Peptides. Pharmaceuticals.

[88] Marín-Medina N., Mescola A., Alessandrini A. (2018). Effects of the Peptide Magainin H2 on Supported Lipid Bilayers Studied by Different Biophysical Techniques. Biochim. Et Biophys. Acta (BBA) Biomembr..

[89] Mescola A., Marín-Medina N., Ragazzini G., Accolla M., Alessandrini A. (2019). Magainin-H2 Effects on the Permeabilization and Mechanical Properties of Giant Unilamellar Vesicles. J. Colloid Interface Sci..

[90] Malanovic N., Lohner K. (2016). Antimicrobial Peptides Targeting Gram-Positive Bacteria. Pharmaceuticals.

[91] Alves I.D., Bechara C., Walrant A., Zaltsman Y., Jiao C.-Y., Sagan S. (2011). Relationships between Membrane Binding, Affinity and Cell Internalization Efficacy of a Cell-Penetrating Peptide: Penetratin as a Case Study. PLoS ONE.

[92] Dupont E., Prochiantz A., Joliot A., Langel Ü. (2011). Penetratin Story: An Overview. Cell-Penetrating Peptides.

[93] Lamazière A., Chassaing G., Trugnan G., Ayala-Sanmartin J. (2009). Tubular Structures in Heterogeneous Membranes Induced by the Cell Penetrating Peptide Penetratin. Commun. Integr. Biol..

[94] Lamazière A., Wolf C., Lambert O., Chassaing G., Trugnan G., Ayala-Sanmartin J. (2008). The Homeodomain Derived Peptide Penetratin Induces Curvature of Fluid Membrane Domains. PLoS ONE.

[95] Almeida C., Lamazière A., Filleau A., Corvis Y., Espeau P., Ayala-Sanmartin J. (2016). Membrane Re-Arrangements and Rippled Phase Stabilisation by the Cell Penetrating Peptide Penetratin. Biochim. Et Biophys. Acta (BBA) Biomembr..

[96] Allolio C., Magarkar A., Jurkiewicz P., Baxová K., Javanainen M., Mason P.E., Šachl R., Cebecauer M., Hof M., Horinek D. (2018). Arginine-Rich Cell-Penetrating Peptides Induce Membrane Multilamellarity and Subsequently Enter via Formation of a Fusion Pore. Proc. Natl. Acad. Sci. USA.

[97] Cardoso A.M.S., Trabulo S., Cardoso A.L., Lorents A., Morais C.M., Gomes P., Nunes C., Lúcio M., Reis S., Padari K. (2012). S4(13)-PV Cell-Penetrating Peptide Induces Physical and Morphological Changes in Membrane-Mimetic Lipid Systems and Cell Membranes: Implications for Cell Internalization. Biochim. Et Biophys. Acta (BBA) Biomembr..

[98] Hassan-Zadeh E., Hussain F., Huang J. (2017). Gramicidin Peptides Alter Global Lipid Compositions and Bilayer Thicknesses of Coexisting Liquid-Ordered and Liquid-Disordered Membrane Domains. Langmuir.

[99] Mescola A., Ragazzini G., Alessandrini A. (2020). Daptomycin Strongly Affects the Phase Behavior of Model Lipid Bilayers. J. Phys. Chem. B.

[100] Su J., Marrink S.J., Melo M.N. (2020). Localization Preference of Antimicrobial Peptides on Liquid-Disordered Membrane Domains. Front. Cell Dev. Biol..

[101] Lee M.-T., Hung W.-C., Hsieh M.-H., Chen H., Chang Y.-Y., Huang H.W. (2017). Molecular State of the Membrane-Active Antibiotic Daptomycin. Biophys. J..

[102] Balleza D., Mescola A., Alessandrini A. (2020). Model Lipid Systems and Their Use to Evaluate the Phase State of Biomembranes, Their Mechanical Properties and the Effect of Non-Conventional Antibiotics: The Case of Daptomycin. Eur. Biophys. J..

[103] Huang H.W. (2020). DAPTOMYCIN, Its Membrane-Active Mechanism vs. That of Other Antimicrobial Peptides. Biochim. Et Biophys. Acta (BBA) Biomembr..

[104] Alonso M.A., Millán J. (2001). The Role of Lipid Rafts in Signalling and Membrane Trafficking in T Lymphocytes. J. Cell Sci..

[105] Ikonen E. (2001). Roles of Lipid Rafts in Membrane Transport. Curr. Opin. Cell Biol..

[106] Krause M.R., Regen S.L. (2014). The Structural Role of Cholesterol in Cell Membranes: From Condensed Bilayers to Lipid Rafts. Acc. Chem. Res..

[107] Liscum L., Dahl N. (1992). Intracellular Cholesterol Transport. J. Lipid Res..

[108] Subczynski W.K., Pasenkiewicz-Gierula M., Widomska J., Mainali L., Raguz M. (2017). High Cholesterol/Low Cholesterol: Effects in Biological Membranes: A Review. Cell Biochem. Biophys..

[109] van Meer G., Voelker D.R., Feigenson G.W. (2008). Membrane Lipids: Where They Are and How They Behave. Nat. Rev. Mol. Cell Biol..

[110] Eid J., Razmazma H., Jraij A., Ebrahimi A., Monticelli L. (2020). On Calculating the Bending Modulus of Lipid Bilayer Membranes from Buckling Simulations. J. Phys. Chem. B.

[111] Lorents A., Säälik P., Langel Ü., Pooga M. (2018). Arginine-Rich Cell-Penetrating Peptides Require Nucleolin and Cholesterol-Poor Subdomains for Translocation across Membranes. Bioconjugate Chem..

[112] Brock R. (2014). The Uptake of Arginine-Rich Cell-Penetrating Peptides: Putting the Puzzle Together. Bioconjugate Chem..

[113] Crosio M.A., Via M.A., Cámara C.I., Mangiarotti A., Del Pópolo M.G., Wilke N. (2019). Interaction of a Polyarginine Peptide with Membranes of Different Mechanical Properties. Biomolecules.

[114] Sharmin S., Islam M.Z., Karal M.A.S., Alam Shibly S.U., Dohra H., Yamazaki M. (2016). Effects of Lipid Composition on the Entry of Cell-Penetrating Peptide Oligoarginine into Single Vesicles. Biochemistry.

[115] Pae J., Säälik P., Liivamägi L., Lubenets D., Arukuusk P., Langel Ü., Pooga M. (2014). Translocation of Cell-Penetrating Peptides across the Plasma Membrane Is Controlled by Cholesterol and Microenvironment Created by Membranous Proteins. J. Control. Release.

[116] Caesar C.E.B., Esbjörner E.K., Lincoln P., Nordén B. (2006). Membrane Interactions of Cell-Penetrating Peptides Probed by Tryptophan Fluorescence and Dichroism Techniques: Correlations of Structure to Cellular Uptake. Biochemistry.

[117] Islam M.Z., Sharmin S., Levadnyy V., Alam Shibly S.U., Yamazaki M. (2017). Effects of Mechanical Properties of Lipid Bilayers on the Entry of Cell-Penetrating Peptides into Single Vesicles. Langmuir.

[118] Futaki S., Nakase I. (2017). Cell-Surface Interactions on Arginine-Rich Cell-Penetrating Peptides Allow for Multiplex Modes of Internalization. Acc. Chem. Res..

[119] Stanzl E.G., Trantow B.M., Vargas J.R., Wender P.A. (2013). Fifteen Years of Cell-Penetrating, Guanidinium-Rich Molecular Transporters: Basic Science, Research Tools, and Clinical Applications. Acc. Chem. Res..

[120] Liu B.R., Chiou S.-H., Huang Y.-W., Lee H.-J. (2022). Bio-Membrane Internalization Mechanisms of Arginine-Rich Cell-Penetrating Peptides in Various Species. Membranes.

[121] Murayama T., Masuda T., Afonin S., Kawano K., Takatani-Nakase T., Ida H., Takahashi Y., Fukuma T., Ulrich A.S., Futaki S. (2017). Loosening of Lipid Packing Promotes Oligoarginine Entry into Cells. Angew. Chem. Int. Ed..

[122] Watkins C.L., Schmaljohann D., Futaki S., Jones A.T. (2009). Low Concentration Thresholds of Plasma Membranes for Rapid Energy-Independent Translocation of a Cell-Penetrating Peptide. Biochem. J..

[123] Neundorf I., Matsuzaki K. (2019). Antimicrobial and Cell-Penetrating Peptides: How to Understand Two Distinct Functions Despite Similar Physicochemical Properties. Antimicrobial Peptides.

[124] MacCallum J.L., Bennett W.F.D., Tieleman D.P. (2008). Distribution of Amino Acids in a Lipid Bilayer from Computer Simulations. Biophys. J..

[125] Li J., Koh J.-J., Liu S., Lakshminarayanan R., Verma C.S., Beuerman R.W. (2017). Membrane Active Antimicrobial Peptides: Translating Mechanistic Insights to Design. Front. Neurosci..

[126] Edwards-Gayle C.J.C., Barrett G., Roy S., Castelletto V., Seitsonen J., Ruokolainen J., Hamley I.W. (2020). Selective Antibacterial Activity and Lipid Membrane Interactions of Arginine-Rich Amphiphilic Peptides. ACS Appl. Bio Mater..

[127] Almeida C.V., de Oliveira C.F.R., dos Santos E.L., dos Santos H.F., Júnior E.C., Marchetto R., da Cruz L.A., Ferreira A.M.T., Gomes V.M., Taveira G.B. (2021). Differential Interactions of the Antimicrobial Peptide, RQ18, with Phospholipids and Cholesterol Modulate Its Selectivity for Microorganism Membranes. Biochim. Et Biophys. Acta (BBA) Gen. Subj..

[128] Li J., Lu X., Ma W., Chen Z., Sun S., Wang Q., Yuan B., Yang K. (2021). Cholesterols Work as a Molecular Regulator of the Antimicrobial Peptide-Membrane Interactions. Front. Mol. Biosci..

[129] Sood R., Kinnunen P.K.J. (2008). Cholesterol, Lanosterol, and Ergosterol Attenuate the Membrane Association of LL-37(W27F) and Temporin L. Biochim. Et Biophys. Acta (BBA) Biomembr..

[130] Henderson J.M., Iyengar N.S., Lam K.L.H., Maldonado E., Suwatthee T., Roy I., Waring A.J., Lee K.Y.C. (2019). Beyond Electrostatics: Antimicrobial Peptide Selectivity and the Influence of Cholesterol-Mediated Fluidity and Lipid Chain Length on Protegrin-1 Activity. Biochim. Et Biophys. Acta (BBA) Biomembr..

[131] Ishitsuka Y., Pham D.S., Waring A.J., Lehrer R.I., Lee K.Y.C. (2006). Insertion Selectivity of Antimicrobial Peptide Protegrin-1 into Lipid Monolayers: Effect of Head Group Electrostatics and Tail Group Packing. Biochim. Et Biophys. Acta (BBA) Biomembr..

[132] Losada-Pérez P., Khorshid M., Hermans C., Robijns T., Peeters M., Jiménez-Monroy K.L., Truong L.T.N., Wagner P. (2014). Melittin Disruption of Raft and Non-Raft-Forming Biomimetic Membranes: A Study by Quartz Crystal Microbalance with Dissipation Monitoring. Colloids Surf. B Biointerfaces.

[133] Prenner E.J., Lewis R.N.A.H., Jelokhani-Niaraki M., Hodges R.S., McElhaney R.N. (2001). Cholesterol Attenuates the Interaction of the Antimicrobial Peptide Gramicidin S with Phospholipid Bilayer Membranes. Biochim. Et Biophys. Acta (BBA) Biomembr..

[134] Sani M.-A., Whitwell T.C., Separovic F. (2012). Lipid Composition Regulates the Conformation and Insertion of the Antimicrobial Peptide Maculatin 1.1. Biochim. Et Biophys. Acta (BBA) Biomembr..

[135] Mason A.J., Marquette A., Bechinger B. (2007). Zwitterionic Phospholipids and Sterols Modulate Antimicrobial Peptide-Induced Membrane Destabilization. Biophys. J..

[136] Yeow E.K.L., Clayton A.H.A. (2007). Enumeration of Oligomerization States of Membrane Proteins in Living Cells by Homo-FRET Spectroscopy and Microscopy: Theory and Application. Biophys. J..

[137] Gorbenko G., Trusova V., Donev R. (2011). Protein Aggregation in a Membrane Environment. Advances in Protein Chemistry and Structural Biology.

[138] Bouvier M. (2001). Oligomerization of G-Protein-Coupled Transmitter Receptors. Nat. Rev. Neurosci..

[139] Ullrich A., Schlessinger J. (1990). Signal Transduction by Receptors with Tyrosine Kinase Activity. Cell.

[140] Parton R.G., McMahon K.-A., Wu Y. (2020). Caveolae: Formation, Dynamics, and Function. Curr. Opin. Cell Biol..

[141] Blood P.D., Voth G.A. (2006). Direct Observation of Bin/Amphiphysin/Rvs (BAR) Domain-Induced Membrane Curvature by Means of Molecular Dynamics Simulations. Proc. Natl. Acad. Sci. USA.

[142] Kozlov M.M., Campelo F., Liska N., Chernomordik L.V., Marrink S.J., McMahon H.T. (2014). Mechanisms Shaping Cell Membranes. Curr. Opin. Cell Biol..

[143] Peter B.J., Kent H.M., Mills I.G., Vallis Y., Butler P.J.G., Evans P.R., McMahon H.T. (2004). BAR Domains as Sensors of Membrane Curvature: The Amphiphysin BAR Structure. Science.

[144] Luckey M. (2008). Membrane Structural Biology: With Biochemical and Biophysical Foundations.

[145] Huan Y., Kong Q., Mou H., Yi H. (2020). Antimicrobial Peptides: Classification, Design, Application and Research Progress in Multiple Fields. Front. Microbiol..

[146] Mahlapuu M., Håkansson J., Ringstad L., Björn C. (2016). Antimicrobial Peptides: An Emerging Category of Therapeutic Agents. Front. Cell. Infect. Microbiol..

[147] Mani R., Cady S.D., Tang M., Waring A.J., Lehrer R.I., Hong M. (2006). Membrane-Dependent Oligomeric Structure and Pore Formation of a β-Hairpin Antimicrobial Peptide in Lipid Bilayers from Solid-State NMR. Proc. Natl. Acad. Sci. USA.

[148] Wang S.-H., Wang C.-F., Chang T.-W., Wang Y.-J., Liao Y.-D. (2019). Oligomerization and Insertion of Antimicrobial Peptide TP4 on Bacterial Membrane and Membrane-Mimicking Surfactant Sarkosyl. PLoS ONE.

[149] Chiti F., Dobson C.M. (2017). Protein Misfolding, Amyloid Formation, and Human Disease: A Summary of Progress Over the Last Decade. Annu Rev. Biochem..

[150] Albanese A., Chan W.C.W. (2011). Effect of Gold Nanoparticle Aggregation on Cell Uptake and Toxicity. ACS Nano.

[151] Yang Z., Malinick A.S., Yang T., Cheng W., Cheng Q. (2020). Gold Nanoparticle-Coupled Liposomes for Enhanced Plasmonic Biosensing. Sens. Actuators Rep..

[152] Johannes L., Pezeshkian W., Ipsen J.H., Shillcock J.C. (2018). Clustering on Membranes: Fluctuations and More. Trends Cell Biol..

[153] Goswami D., Gowrishankar K., Bilgrami S., Ghosh S., Raghupathy R., Chadda R., Vishwakarma R., Rao M., Mayor S. (2008). Nanoclusters of GPI-Anchored Proteins Are Formed by Cortical Actin-Driven Activity. Cell.

[154] Jaqaman K., Kuwata H., Touret N., Collins R., Trimble W.S., Danuser G., Grinstein S. (2011). Cytoskeletal Control of CD36 Diffusion Promotes Its Receptor and Signaling Function. Cell.

[155] Raghupathy R., Anilkumar A.A., Polley A., Singh P.P., Yadav M., Johnson C., Suryawanshi S., Saikam V., Sawant S.D., Panda A. (2015). Transbilayer Lipid Interactions Mediate Nanoclustering of Lipid-Anchored Proteins. Cell.

[156] Bassereau P., Sens P. (2018). Physics of Biological Membranes.

[157] Helfrich W. (1973). Elastic Properties of Lipid Bilayers: Theory and Possible Experiments. Z. Für Nat. C.

[158] Kim K.S., Neu J., Oster G. (1998). Curvature-Mediated Interactions Between Membrane Proteins. Biophys. J..

[159] Auth T., Gompper G. (2009). Budding and Vesiculation Induced by Conical Membrane Inclusions. Phys. Rev. E.

[160] Deserno M., Kremer K., Paulsen H., Peter C., Schmid F., Basché T., Müllen K., Schmidt M. (2014). Computational Studies of Biomembrane Systems: Theoretical Considerations, Simulation Models, and Applications. From Single Molecules to Nanoscopically Structured Materials.

[161] Goulian M., Bruinsma R., Pincus P. (1993). Long-Range Forces in Heterogeneous Fluid Membranes. EPL.

[162] Dommersnes P.G., Fournier J.B., Galatola P. (1998). Long-Range Elastic Forces between Membrane Inclusions in Spherical Vesicles. Europhys. Lett..

[163] Safouane M., Berland L., Callan-Jones A., Sorre B., Römer W., Johannes L., Toombes G.E.S., Bassereau P. (2010). Lipid Cosorting Mediated by Shiga Toxin Induced Tubulation. Traffic.

[164] Schweitzer Y., Kozlov M.M. (2015). Membrane-Mediated Interaction between Strongly Anisotropic Protein Scaffolds. PLoS Comput. Biol..

[165] Simons K., Gerl M.J. (2010). Revitalizing Membrane Rafts: New Tools and Insights. Nat. Rev. Mol. Cell Biol..

[166] Rossy J., Ma Y., Gaus K. (2014). The Organisation of the Cell Membrane: Do Proteins Rule Lipids?. Curr. Opin. Chem. Biol..

[167] Johannes L., Wunder C., Shafaq-Zadah M. (2016). Glycolipids and Lectins in Endocytic Uptake Processes. J. Mol. Biol..

[168] Anderson R.G.W., Jacobson K. (2002). Cell Biology: A Role for Lipid Shells in Targeting Proteins to Caveolae, Rafts, and Other Lipid Domains. Science.

[169] Marčelja S. (1976). Lipid-Mediated Protein Interaction in Membranes. Biochim. Et Biophys. Acta (BBA) Biomembr..

[170] Sintes T., Baumgärtner A. (1997). Protein Attraction in Membranes Induced by Lipid Fluctuations. Biophys. J..

[171] Haselwandter C.A., Wingreen N.S. (2014). The Role of Membrane-Mediated Interactions in the Assembly and Architecture of Chemoreceptor Lattices. PLoS Comput. Biol..

[172] Morozova D., Guigas G., Weiss M. (2011). Dynamic Structure Formation of Peripheral Membrane Proteins. PLoS Comput. Biol..

[173] Lavagna E., Bochicchio D., Marco A.L.D., Güven Z.P., Stellacci F., Rossi G. (2022). Ion-Bridges and Lipids Drive Aggregation of Same-Charge Nanoparticles on Lipid Membranes. Nanoscale.

[174] Chan H., Král P. (2018). Nanoparticles Self-Assembly within Lipid Bilayers. ACS Omega.

[175] Rasch M.R., Rossinyol E., Hueso J.L., Goodfellow B.W., Arbiol J., Korgel B.A. (2010). Hydrophobic Gold Nanoparticle Self-Assembly with Phosphatidylcholine Lipid: Membrane-Loaded and Janus Vesicles. Nano Lett..

[176] Angelikopoulos P., Sarkisov L., Cournia Z., Gkeka P. (2017). Self-Assembly of Anionic, Ligand-Coated Nanoparticles in Lipid Membranes. Nanoscale.

[177] Lavagna E., Barnoud J., Rossi G., Monticelli L. (2020). Size-Dependent Aggregation of Hydrophobic Nanoparticles in Lipid Membranes. Nanoscale.

[178] Petretto E., Ong Q.K., Olgiati F., Ting M., Campomanes P., Stellacci F., Vanni S. (2021). Ion-Mediated Charge-Charge Interactions Drive Aggregation of Surface-Functionalized Gold Nanoparticles.

[179] Lavagna E., Güven Z.P., Bochicchio D., Olgiati F., Stellacci F., Rossi G. (2021). Amphiphilic Nanoparticles Generate Curvature in Lipid Membranes and Shape Liposome–Liposome Interfaces. Nanoscale.

[180] Gessner I., Neundorf I. (2020). Nanoparticles Modified with Cell-Penetrating Peptides: Conjugation Mechanisms, Physicochemical Properties, and Application in Cancer Diagnosis and Therapy. IJMS.

[181] Chiarpotti M.V., Longo G.S., Del Pópolo M.G. (2021). Nanoparticles Modified with Cell Penetrating Peptides: Assessing Adsorption on Membranes Containing Acidic Lipids. Colloids Surf. B Biointerfaces.

[182] Kim E.Y., Kumar D., Khang G., Lim D.-K. (2015). Recent Advances in Gold Nanoparticle-Based Bioengineering Applications. J. Mater. Chem. B.

[183] Li D., He Q., Li J. (2009). Smart Core/Shell Nanocomposites: Intelligent Polymers Modified Gold Nanoparticles. Adv. Colloid Interface Sci..

[184] Dai Y., Zhang X. (2018). Recent Advances in Amphiphilic Polymers as the Stabilizers of Colloidal Gold Nanoparticles. Macromol. Mater. Eng..

[185] Tahir M.A., Guven Z.P., Arriaga L.R., Tinao B., Yang Y.-S.S., Bekdemir A., Martin J.T., Bhanji A.N., Irvine D., Stellacci F. (2020). Calcium-Triggered Fusion of Lipid Membranes Is Enabled by Amphiphilic Nanoparticles. Proc. Natl. Acad. Sci. USA.

